# Visualization of polymer relaxation in viscoelastic turbulent micro-channel flow

**DOI:** 10.1038/srep16633

**Published:** 2015-11-13

**Authors:** Jiayan Tai, Chun Ping Lim, Yee Cheong Lam

**Affiliations:** 1 School of Mechanical and Aerospace Engineering, Nanyang Technological University, Singapore

## Abstract

In micro-channels, the flow of viscous liquids e.g. water, is laminar due to the low Reynolds number in miniaturized dimensions. An aqueous solution becomes viscoelastic with a minute amount of polymer additives; its flow behavior can become drastically different and turbulent. However, the molecules are typically invisible. Here we have developed a novel visualization technique to examine the extension and relaxation of polymer molecules at high flow velocities in a viscoelastic turbulent flow. Using high speed videography to observe the fluorescein labeled molecules, we show that viscoelastic turbulence is caused by the sporadic, non-uniform release of energy by the polymer molecules. This developed technique allows the examination of a viscoelastic liquid at the molecular level, and demonstrates the inhomogeneity of viscoelastic liquids as a result of molecular aggregation. It paves the way for a deeper understanding of viscoelastic turbulence, and could provide some insights on the high Weissenberg number problem. In addition, the technique may serve as a useful tool for the investigations of polymer drag reduction.

Viscoelastic liquids consist of macro-molecules i.e. polymers, typically suspended in a solvent. In our daily lives, the flow of viscoelastic liquids in confined conduits is prevalent e.g. air-conditioning chillers, waste water and crude oil pipelines, hemodynamics etc. At low Reynolds numbers (Re < 1), these liquids can generate flow instabilities in the absence of inertial forces and cause the usual laminar flow in the micro-channel to become turbulent, i.e. viscoelastic turbulence^1^. Under such conditions, the Deborah number (De), which describes the relative importance of elastic forces to viscous forces, takes precedence as the critical parameter that governs the stability of the flow field^2^. Over the years, viscoelastic instabilities have been reported in many configurations e.g. contraction-expansion flows^2^,^3^, cross-slot flows^4^,^5^, T-channel flows^6^, and Taylor-Couette flows^7^,^8^. The possibility of generating turbulence at low Re also motivated rapid progress in micro-channel mixing applications^2^,^9^,^10^,^11^,^12^.

It is fundamental to understand how these small quantities of polymer molecules, which cause the solution to become viscoelastic, could result in such significant changes to the flow behavior. However, the molecules are typically invisible, and the bulk behavior of a viscoelastic solution is usually deduced by observing the motion of tracer additives, with the implicit assumption that the solution is homogenous. It would be interesting to directly observe the behavior of polymer molecules causing the turbulent flow. Such observations may also provide insights on the underlying mechanisms of polymer drag reduction^13^ in energy conservation applications e.g. oil pipelines^14^ and air-conditioning systems^15^ etc.

To gain insights on the behavior of the invisible polymer molecules in viscoelastic flows, some studies have employed the use of fluorescently stained DNA probes^16^,^17^,^18^,^19^. The probes were seeded into the viscoelastic liquids, which consisted of unstained monodispersed^16^,^19^ or polydispersed^16^,^17^,^18^ polymer molecules. Besides observing the conformations of the DNA probes, the molecular extensions were measured and used to deduce the elastic stresses^19^ generated by the unstained monodispersed DNA molecules. However, the stained tracer molecules have different contour lengths and relaxation properties^20^, and are not representative of the host molecules. In addition, the DNA probes and monodispersed DNA viscoelastic liquids are costly for the investigation of large scale flows.

Here, we present a high-speed molecular imaging technique for the visualization of molecular conformation changes, i.e. stretching and relaxation, which are directly related to the amount of stored elastic energy in the polymer molecules. This technique involves fluorescein labeling of polymer molecules so that they appear visible under illumination tailored to the specific wavelength of the fluorescent dye. Using a high speed camera to capture images at up to 1,000 frames per second (FPS), polymer molecular conformations at high flow velocities can be observed directly. The molecular conformation changes can be correlated to the state of the flow field, i.e. laminar or turbulent flow, which was quantified using the conventional seeding method via Particle Image Velocimetry (PIV).

In contrast to the studies based on DNA probes, the polymer molecules in the current study do not act as probes for elastic stresses; they are very much part of the solutions. As such, observations were made directly on the solution component, which is the cause of its elastic nature. In addition, the conformation changes in DNA probes were studied at much lower recording rates (up to 100 FPS^18^), and the high speed observations attained in the current study has not been reported. More importantly, it should be highlighted that the current study is on the investigation of a typical and representative polydispersed viscoelastic polymeric liquid.

## Results

### Flow configuration

A 3-stream contraction-expansion micro-channel design^2^ was employed for the investigation. The inlet consists of a highly viscoelastic main-stream liquid, enveloped by two side-stream liquids of lower elasticity. This configuration was reported to produce chaotic flow and enhanced mixing. Supplementary Fig. S1 shows the 2-D schematic diagram of the micro-channel. Here, the investigation focuses on the flow field downstream of the contraction where turbulence occurs.

### Test fluids

Polymer molecules were labeled with a fluorescent dye i.e. fluorescein isothiocyanate (FITC), so that they were visible when illuminated at the excitation wavelength of the dye. Polyacrylamide (PAA, 5 × 10^6^–6 × 10^6^ g/mol, Sigma Aldrich) was used as the base polymer. PAA was modified and labeled with FITC molecules along its backbone to obtain fluorescein-labeled partially hydrolyzed polyacrylamide (phPAA-FITC), via a fluorescein labeling procedure (refer to Methods section). This provided high enough intensity for the polymer molecules to be observed under high flow velocities. The main-stream and side-stream liquids consisted of 1.5 wt% and 0.1 wt% phPAA-FITC in DI water respectively. To facilitate the conventional PIV measurements, polystyrene particles (5 μm, Thermo Scientific) were seeded to the liquids (0.1 wt%), and the flow field was quantified in a separate experiment. Experimental runs at two representative flow rates are presented i.e. 1 ml/h (Re = 0.003, De = 41.46, 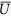 = 0.014 m/s) and 10 ml/h (Re = 0.217, De = 414.6, 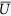 = 0.146 m/s). Here, 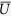 represents the mean velocity of the main-stream liquid in the contraction. The Raman spectroscopy tests, fluid rheological properties and calculation of flow parameters i.e. Re and De, have been included in the Supplementary Information (Supplementary Fig. S2; Supplementary Table S1; Supplementary Notes).

### Experimental details

**Figure 1:**
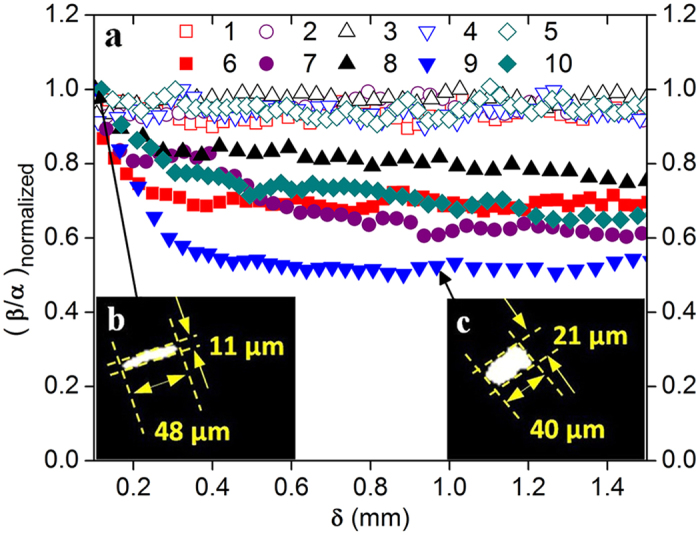
Comparative analyses of the polymer stresses released at low and high Deborah numbers. (**a**) Polymer relaxation, represented by the normalized ratio of the major-axis (β) to the minor-axis (α), over axial distance (δ = 0.1–1.5 mm). β and α were obtained by fitting an ellipse using a MATLAB algorithm. Open and closed symbols represent polymers captured at De = 41.46 and 414.6 respectively. Significant relaxation occurs at De = 414.6 only. Inserts show the relaxation of polymer molecule 9 at δ = 0.1 mm (**b**) and 1.0 mm (**c**) respectively, with a reduction in area (=33.6%), at De = 414.6.

The conformations of the phPAA-FITC molecules were dynamically tracked under epi-fluorescence illumination at De = 41.46 (1 ml/h) and 414.6 (10 ml/h). Here, the observed phPAA-FITC molecules were polymer aggregates; this occurs naturally even at dilute concentrations^21^,^22^. Details of the molecular imaging setup have been included in the Methods section. To avoid any blurring effects near the contraction, analyses of the polymer molecules were only carried out from some distance (δ = 0.1–1.5 mm) away from the contraction, with δ defined in Supplementary Fig. S1. An ellipse fitting algorithm was applied on the polymer molecules to obtain the major-axis (β) and minor-axis (α) respectively. Here, the aspect ratio of the polymer molecule is defined as β/α. To observe the degree of relaxation of the molecules, (β/α)_normalized_ was obtained by normalizing the aspect ratio at each δ, with that at δ = 0.1 mm. Hence, a larger change in (β/α)_normalized_ corresponds to a larger release of stored elastic energy. Figure 1 shows the normalized aspect ratio i.e. (β/α)_normalized_, of phPAA-FITC molecules with respect to δ, at De = 41.46 and De = 414.6 respectively. For clarity, only 5 phPAA-FITC molecules were plotted for each flow rate. Separately, conventional PIV was also employed to quantify the flow field, and to facilitate the correlation of the polymer conformation changes to the state of the flow field. Details of the PIV measurements have been included in the Supplementary Notes.

### Aspect ratio

**Figure 2:**
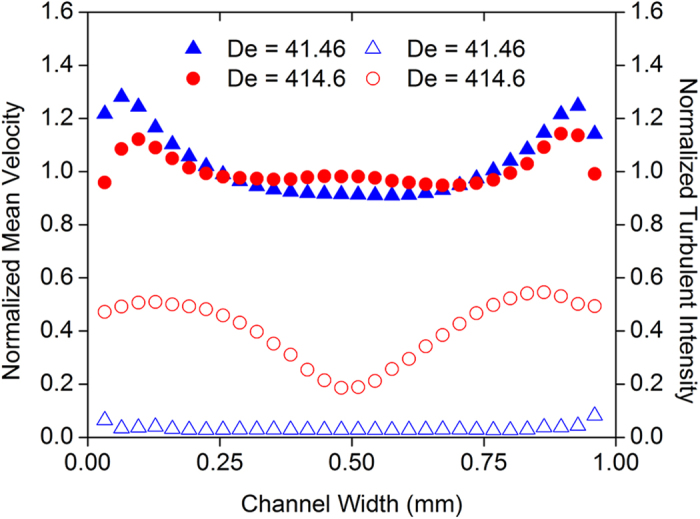
Normalized mean velocity profiles and turbulent intensities. Data were obtained at δ = 0.3 mm for De = 41.46 and 414.6. Closed symbols correspond to velocity profiles while open symbols correspond to turbulent intensities. The velocity profile exhibits a central peak for De = 414.6, providing evidence that the fluid has not relaxed completely. Such high turbulent intensities observed at De = 414.6 are typically only reported at high Reynolds numbers^23^,^24^.

At De = 41.46, no significant changes in molecule aspect ratio can be observed and (β/α)_normalized_ fluctuates about unity (≈0.9–1, Fig. 1). This indicates little stretching of the molecules in the contraction; there is negligible relaxation of the polymer molecules and hence insignificant release of stored elastic energy subsequently. As expected, the PIV results indicate a laminar flow state with negligible turbulent intensities (Fig. 2).

**Figure 3:**
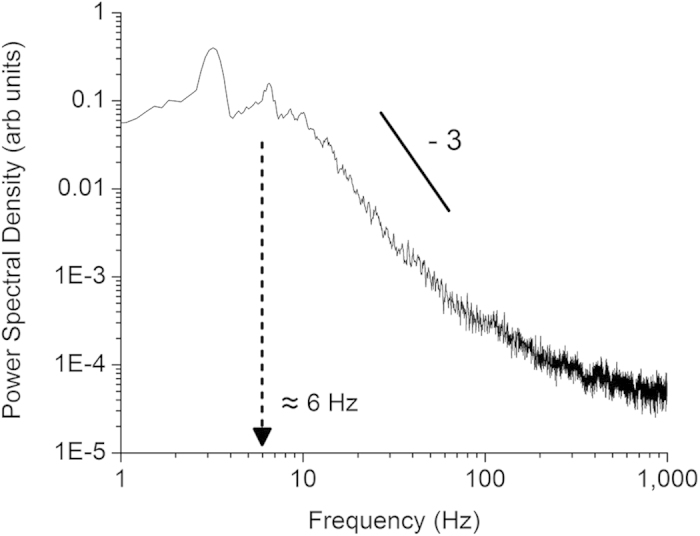
Power law decay in viscoelastic turbulence. Power spectrum of the velocity fluctuations at δ = 0.3 mm along the channel mid-line (see Supplementary Fig. S1) at De = 414.6. The data were acquired at 2000 Hz over 38 seconds. Short recording times resulted in few data points below 1 Hz, hence only data above 1 Hz are shown. The decay originates at ≈6 Hz, close to the characteristic time (1/λ_e_ = 3.76 Hz, where λ_e_ is the extensional relaxation time, see Supplementary Information) of the main-stream fluid i.e. an indication that ‘large eddies’ are dependent on the fluid relaxation properties. This is in contrast to the previous experimental observations^1^,^9^, where power law decays originate at lower frequencies, i.e. <1 Hz.

In contrast, at De = 414.6, a significant decrease in molecule aspect ratio can be observed, whereby (β/α)_normalized_ reduces to a range of ≈0.5–0.8 at the end of the recording window (Fig. 1). This indicates that the molecules were significantly stretched in the contraction, which allowed subsequent relaxation of the molecules with a corresponding release of the stored elastic energy. The molecule relaxation occurred randomly and sporadically. This resulted from the inhomogeneity of the bulk liquid due to the formation of polymer aggregates, which were also not uniformly distributed in the solution. In addition, the extent and rate of relaxation varied among the individual molecules (Fig. 1). These caused the flow field to become turbulent, characterized by high turbulent intensities (Fig. 2), which were not observed when Newtonian liquids were used (refer to Supplementary Fig. S3 and Supplementary Notes). Such turbulent characteristics are typically only observed at high Reynolds numbers in large channels^23^,^24^. Furthermore, the power spectra of the axial velocity fluctuations revealed a power law decay with a slope ≈−3 (Fig. 3). This is quantitatively similar to the observations reported in literature^1^,^9^ for viscoelastic turbulent flows.

At De = 414.6, most of the molecular relaxation occurs before δ = 0.4 mm (Fig. 1). This agrees well with the PIV results showing a diminished peak in the mean velocity profile at δ = 0.3 mm, indicating the almost complete release of elastic stresses in the main-stream liquid (Fig. 2). It should be highlighted that the extensional stresses in the molecules have almost been completely released at δ = 1.4–1.5 mm, because (β/α)_normalized_ reached a stabilized value and does not change significantly beyond δ = 0.6 mm (see Fig. 1). For both flow rates (De = 41.46 and 414.6), the average aspect ratio approached a similar value (β/α = 1.8) at δ = 1.4–1.5 mm, which is 20% higher than that of molecules measured under static conditions (β/α = 1.5). This is due to molecular stretching as a result of shear stresses within the flow. The static measurements of the unstressed phPAA-FITC molecules have been included in the Methods section and Supplementary Information (Supplementary Fig. S4; Supplementary Table S2).

### Molecular size

The captured images of a phPAA-FITC molecule (see Fig. 1, molecule 9) at De = 414.6 are included in Fig. 1b (δ = (P0.1 mm) and Fig. 1c (δ = (P1.0 mm) respectively. Due to the extensional stresses in the contraction, the observed area of the polymer molecule decreased 33.6% on exit from the contraction as compared to the average area at δ = 1.4–1.5 mm. Details of the computation can be found in the Methods section. We further analyzed 20 molecules, each at De = 41.46 and 414.6 respectively, and found the area of each molecule decreased by an average of 4.8% in the former, and 15.1% in the latter.

## Discussion

The changes in molecular area and (β/α)_normalized_ observed can be directly attributed to the release of stored elastic energy in the stretched polymer molecules. The much larger change in area and aspect ratio at De = 414.6 is due to the larger extensional stresses at this higher flow rate in the contraction, which resulted in significant release of elastic energy subsequently. Thus, the non-uniform and sporadic release of energy by the polymer molecules resulted in turbulent flow, showing that viscoelastic solutions are not homogenous as dictated by their bulk properties. This inhomogeneity effect occurs as a result of molecular aggregation, and is typically neglected in the investigations and modeling of viscoelastic liquid flows. For an in-depth understanding of the flow dynamics of a viscoelastic solution, the aggregation of polymer molecules could be important.

In summary, many liquids are viscoelastic e.g. blood, mucus, polymeric suspensions, surfactants etc. Understanding the role of the elastic component of the liquid, i.e. the polymer molecules in this investigation, will pave the way for an in-depth understanding and explanation of viscoelastic turbulence. In addition, the developed technique could be used as a tool for investigations on polymer drag reduction, and provide insights on alleviating the high Weissenberg number problem^25^.

## Methods

### Micro-channel design

Supplementary Fig. S1 shows a schematic diagram of the micro-channel, which was fabricated by etching a bottom silicon layer using Deep Reactive Ion Etching (DRIE), and subsequently bonding it to a top Pyrex glass layer via anodic bonding. The micro-channel consists of 3 inlets and 1 outlet. The test liquids were pumped into the micro-channel by high force precision pumps (KDS-410) via glass syringes (Hamilton Gastight). The main-stream liquid (black) is highly viscoelastic, while the side-stream liquid (blue) has lower elasticity. As the liquids flowed towards the outlet, they were forced to accelerate through a sudden contraction (8:1:8). The micro-channel has a depth of 180 μm, and PIV measurements were taken at the mid-plane i.e. 90 μm.

### Fluorescein labeling

Fluorescein isothiocyanate isomer I (FITC, Sigma Aldrich, Product number F7250, λ_excitation_ = 492 nm, λ_emission_ = 518 nm) is a molecule that emits fluorescence at the specified excitation wavelength, and consists of an isothiocyanate group. It has been reported that isothiocyanate groups react with nucleophilic (OH) groups, and FITC was successfully tagged to PEO^26^,^27^. However, there is only one hydroxyl group at each PEO chain end; thus, observing the conformation of the FITC labeled PEO molecule would not be possible. In addition, the molecule would not be able to emit sufficient fluorescence for high speed visualization. Hence, partially hydrolyzed polyacrylamide (phPAA), which has more nucleophilic bonding sites for FITC labeling along the molecular chain, was chosen as a suitable target. phPAA was obtained by alkaline hydrolysis of polyacrylamide (PAA, 5 × 10^6^–6 × 10^6^ g/mol, Sigma Aldrich, Product number 92560).

Firstly, PAA (5 g) was dissolved in DI water (100 g) and gently stirred for 5 hours. Next, hydrolysis was achieved by adding 35.2 g of sodium hydroxide stock solution (2 g of NaOH in 50 g of H_2_O) to the PAA mixture at 60 °C, while gently stirred for 48 hours to ensure a maximum degree of hydrolysis^28^. This concentration was calculated based on a 1:2 ratio between NaOH and the maximum number of amide groups that can be hydrolyzed in PAA, i.e. 50% hydrolysis. Ammonia gas was released as a reaction byproduct (detected with a pH indicator). The resulting phPAA was subsequently extracted via precipitation (1 ml phPAA mixture to 10 ml of anhydrous ethanol). This precipitation step can be repeated until the desired amount of phPAA is obtained. The phPAA was dried at 50 °C, and kept in a desiccator at 30% relative humidity for 2 days.

Next, phPAA was dissolved in DI water (1 wt% phPAA) and gently stirred for 5 hours. The mixture was subsequently cooled down to 4 °C, and 2 parts of fluorescein isothiocyanate stock solution (100 mg of FITC in 10 ml of anhydrous ethanol) was added to 10 parts of the phPAA mixture. The mixture was gently stirred and incubated in the dark for more than 8 hours. During the incubation, carboxyl and hydroxyl groups reacted with isothiocyanate groups to form thiourethane links with the fluorescein. The tagged phPAA molecules (phPAA-FITC) were subsequently extracted via a second precipitation step (1 ml phPAA-FITC mixture to 10 ml of anhydrous ethanol). Similarly, this precipitation step can also be repeated until the desired amount of phPAA-FITC is obtained. The precipitant was rinsed with anhydrous ethanol to remove excess free FITC molecules, and stored in a desiccator at 30% relative humidity for 7 days.

### Raman Spectroscopy

PAA, phPAA, and phPAA-FITC were dissolved in DI water and cast onto a flat aluminum film mounted on a glass slide. The films were then kept in a desiccator at 30% relative humidity for 7 days, to remove excess water. A Raman microscope (Renishaw inVia) with a grating spacing of 1800 lines/mm was used to obtain the Raman spectrum via a HeNe laser source (P = 50 W max, λ = 633 nm). The laser was focused onto the polymer films using a 50X objective lens (Leica Plan Epi, NA = 0.75), and the resulting spectrum was obtained using a laser power of 25 W, over 5 accumulations.

Supplementary Fig. S2 shows the normalized spectra for PAA, phPAA, and phPAA-FITC. A multipoint baseline routine was used to remove background signal, and the spectra was normalized to the CH_2_ Raman band (2900–2940 cm^−1^). In comparison to the PAA spectrum, the phPAA spectrum showed a drop in the amide I band (C = O stretch, 1550–1770 cm^−1^), and attenuation of the peaks at the amide A band (N-H stretch, ≈3190 cm^−1^) and amide B band (N-H stretch, ≈3340 cm^−1^). These changes are due to hydrolysis. As compared to the phPAA spectrum, the phPAA-FITC spectrum showed a rise in the C = S band (C = S stretch, ≈1180 cm^−1^), and a drop in the peak of the O-H band (O-H stretch, ≈3420 cm^−1^). In addition, no peaks could be observed in the isothiocyanate band (stretch, 2020–2100 cm^−1^) of the phPAA-FITC spectrum. These provide evidence of successful reaction between the FITC and phPAA molecule. Hence, by exciting the FITC molecule at the appropriate wavelength i.e. λ_excitation_ = 492 nm, the phPAA-FITC molecule having multiple FITC groups along its molecular chain, can then be observed.

### Molecular imaging setup

A monochrome high speed camera (Photron Fastcam SA5, 32 Gb, sensor pixel size = 20 × 20 μm), was used to capture 8-bit sequential images of the micro-channel flow. The micro-channel was placed on a Nikon microscope (Nikon Eclipse Ti), and illuminated by a white mercury light source. A 10X Nikon microscope lens (Plan Fluor, NA = 0.17, DOF ≈9 μm) was used for magnification, such that one image pixel corresponds to 2 × 2 μm.

The phPAA molecules could not be detected under white light. For their observation, the Nikon B-2A filter configuration (λ_excitation_ = 450–490 nm, λ_emission_ = 500 nm) which corresponds closely to the FITC excitation and emission wavelengths, was used. The recording speeds at De = 41.46 and De = 414.6 were 125 FPS and 1000 FPS respectively. Only polymer molecules that remained in focus were examined, while out of focus data were discarded. Exposure times were set to 0.2 ms. For analyses of molecular conformations, a MATLAB algorithm was used to fit an ellipse onto the perimeter of the polymer molecules, and the major axes (β) and minor axes (α) were obtained for calculating the aspect ratio i.e. β/α. Note that no particles were added to the viscoelastic liquids for the aspect ratio analyses.

### Molecular size measurement

To avoid any blurring effects due to the high speed of the polymer molecules upon exiting the contraction, analyses were only carried out from δ = 0.1–1.5 mm. Based on measurements of the relaxed polymer molecules i.e. from δ = 1.4–1.5 mm, the nominal sizes of the polymer molecules were 33 × 18 μm (β/α = 1.8). This is close to the average size of the unstressed polymer molecule, i.e. 32 × 22 μm (β/α = 1.5), which was obtained by measuring ≈1000 polymer molecules under static conditions. It should be noted that the size of a single PAA molecule^29^ (5 × 10^6^ g/mol) is ≈1–2 μm, and the larger molecular size observed is the result of molecular aggregation^21^,^22^. Supplementary Table S2 shows the size distribution of the molecules, which has been subcategorized into 100 μm^2^ incremental size ranges. Within each size range, the number of counts has been included, together with the average and standard deviation of the size, major-axes and minor-axes. The average aspect ratio i.e. β/α = 1.5, does not vary significantly across the various size ranges. A histogram plot of the distribution is shown in Supplementary Fig. S4.

Subsequently, (β/α)_normalized_ was calculated by normalizing the aspect ratio at each δ, with that at δ = 0.1 mm. For each flow rate, (β/α)_normalized_ is largest at δ = 0.1 mm because of the highly extended state of the polymer upon exiting the contraction, i.e. highest stored elastic energy. The area change in molecules was computed based on (A_final_ − A_initial_)/(A_final_), whereby A_final_ was the average area (obtained from the ellipse fitting algorithm) computed from δ = 1.4–1.5 mm, and A_initial_ was computed near the contraction from δ = 0.1–0.2 mm.

## Additional Information

**How to cite this article**: Tai, J. *et al.* Visualization of polymer relaxation in viscoelastic turbulent micro-channel flow. *Sci. Rep.* **5**, 16633; doi: 10.1038/srep16633 (2015).

## Supplementary Material

Supplementary Information
