# Supplementary material for: Visualization of polymer relaxation in viscoelastic turbulent micro-channel flow
Source: Sci Rep. 2015 Nov 13;5:16633. doi: 10.1038/srep16633 (PMC4643225; doi:10.1038/srep16633)
Supplement: Supplementary Information [file srep16633-s1.pdf]

Supplementary Information for

Visualization of polymer relaxation in viscoelastic turbulent micro-channel flow

Authors: J. Tai, C. P. Lim, Y. C. Lam

Correspondence to: MYClam@ntu.edu.sg

This document includes:

Supplementary Figures S1-S4

Supplementary Tables S1-S2

Supplementary Notes

Supplementary Figures

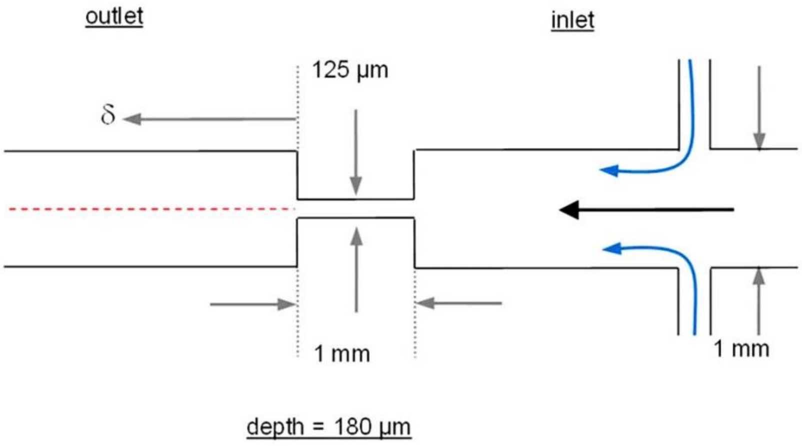

Supplementary Figure S1. Schematic 2-D diagram of the 8:1:8 contraction expansion micro-channel. Arrows depict direction of flow (black = main-stream, blue = side-stream). Red dotted line denotes channel mid-line. δ denotes the distance away from the contraction.

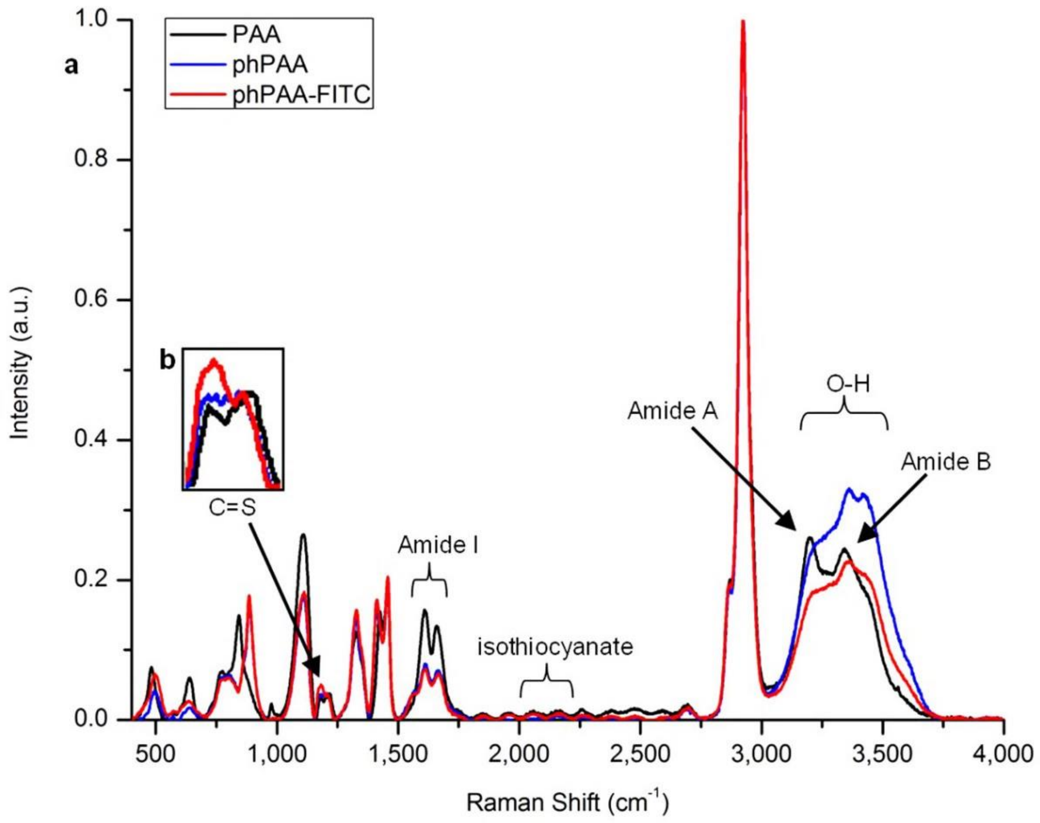

Supplementary Figure S2. Raman Spectroscopy. (**a**) Raman spectrum of PAA, phPAA and phPAA-FITC. Significant bandwidth differences are labeled. The insert (**b**) shows an enlarged view of the C=S band.

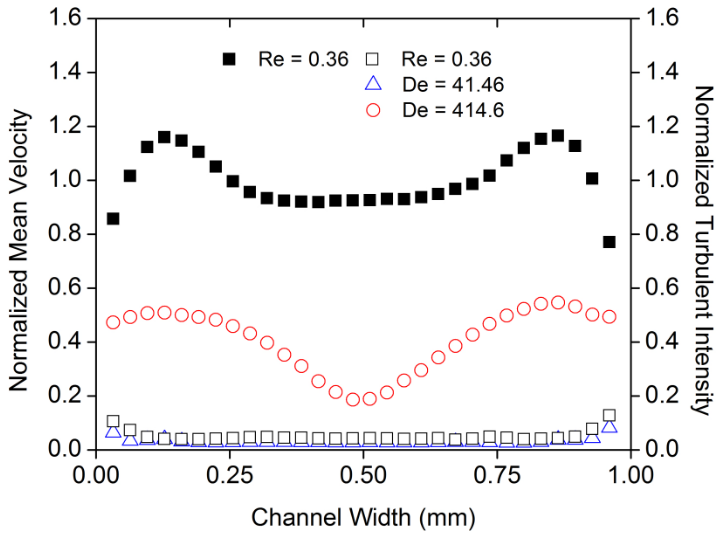

Supplementary Figure S3. Normalized mean velocity profiles and turbulent intensities of Newtonian liquids. Data was obtained at δ = 0.3 mm for Re = 0.36. Closed symbols correspond to velocity profiles while open symbols correspond to turbulent intensities. Note that the velocity profile for Re = 0.36 does not exhibit any central peak, providing evidence that the observed peak of the viscoelastic liquids at Re = 0.217 (De = 414.6, 10 ml/h) in Fig. 2 of the main manuscript was not due to fluid inertia. For comparison, the turbulent intensities at De = 41.46 and De = 414.6 have been included. At De = 414.6, the turbulent intensities were approximately an order of magnitude higher.

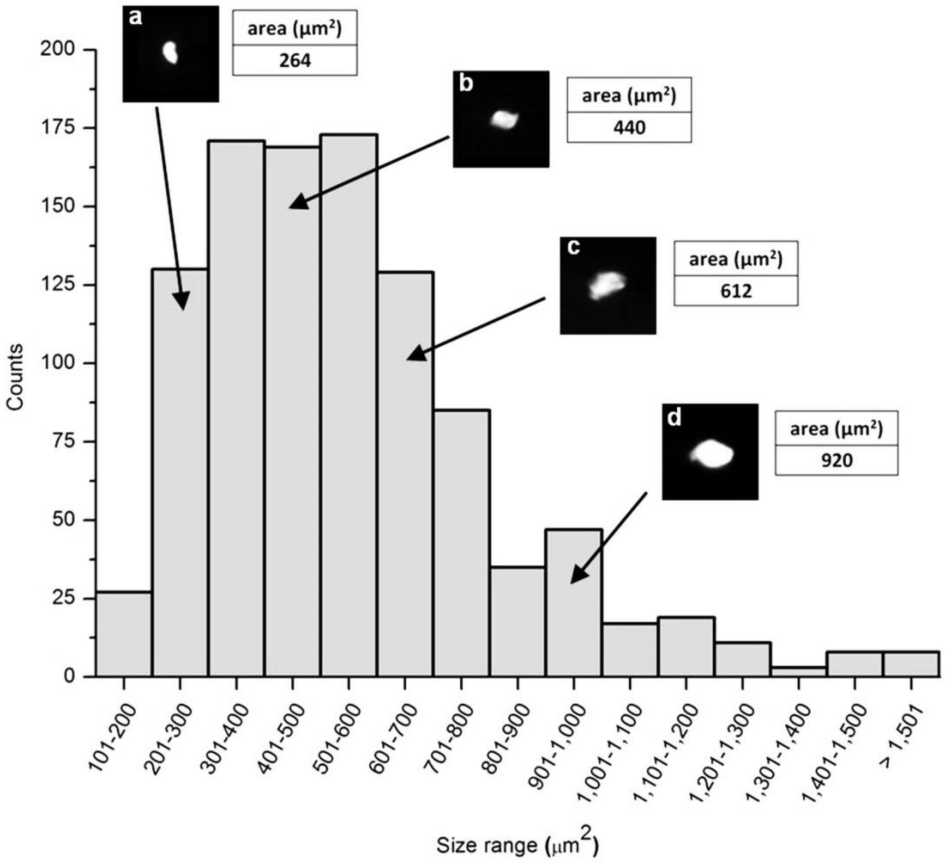

Supplementary Figure S4. Size distribution of phPAA-FITC molecules. Histogram plot of the size ranges for 1000 phPAA-FITC molecules measured under static conditions. Inserts (a-d) show the snapshots of four molecules, with the corresponding area included.

Supplementary Table S1. Rheological properties of test liquids and dimensionless numbers. The average shear rate in the contraction was computed, and the corresponding viscosity was then obtained from the rheological tests for the calculation of the corresponding Reynolds number (Re) and Deborah number (De).

Properties / Fluid	Extensional relaxation time (s)	Flow rate (ml/h)	Contraction shear rate (s^{-1})	Contraction viscosity (Pa.s)	Contraction mean velocity (m/s)	Re	De
Main-stream Liquid	0.266 ± 0.018	1	154	0.374	0.014	0.003	41.46
		10	1718	0.055	0.146	0.217	414.6
Side-stream Liquid	0.019 ± 0.003	1	623	0.008	0.011	0.086	2.327
		10	5710	0.003	0.103	2.241	23.27

Supplementary Table S2. Size distribution of 1000 phPAA-FITC molecules measured under static conditions. The number of counts for the distribution has been plotted in Supplementary Fig. S4. The average aspect ratio is 1.5, and does not vary significantly across the size ranges.

Size range (μm)^2	Size (μm)^2		Counts	Major axes, β (μm)		Minor axes, α (μm)		Mean aspect ratio, β/α
	Average	Standard deviation		Average	Standard deviation	Average	Standard deviation	
101-200	167.70	21.42	27	18.91	3.01	15.10	1.62	1.54
201-300	256.65	29.35	130	23.65	3.85	15.03	2.11	1.63
301-400	349.75	29.91	171	26.84	4.05	17.82	2.33	1.56
401-500	449.49	29.33	169	29.72	3.46	20.38	1.99	1.49
501-600	550.89	28.73	173	32.20	3.66	22.77	2.27	1.44
601-700	645.30	25.88	129	35.14	4.09	24.54	2.42	1.46
701-800	746.45	25.83	85	37.34	5.27	26.72	2.52	1.43
801-900	850.86	33.45	35	40.09	4.84	28.45	2.83	1.44
901-1000	940.68	25.83	47	43.45	7.66	29.59	3.43	1.52
1001-1100	1047.29	31.44	17	46.98	5.32	29.83	3.09	1.61
1101-1200	1137.68	29.25	19	48.40	6.84	31.91	3.44	1.55
1201-1300	1240.00	25.42	11	49.75	12.07	35.29	5.63	1.52
1301-1400	1350.67	37.17	3	48.54	3.44	37.50	4.02	1.31
1401-1500	1436.50	20.45	8	57.40	10.43	35.59	4.89	1.67
> 1501	1802.50	363.68	8	59.96	8.33	41.92	5.57	1.44

Supplementary Notes

Control Experiments (Newtonian Liquids)

As a control measure, a similar set of experiments was conducted using Newtonian liquids in the same micro-channel. The composition of the main-stream and side-stream liquids was 50 wt% glycerol (Sigma Aldrich, Product number G7757) in DI water, and 25 wt% glycerol in DI water respectively. The flow state was laminar and characterized by low turbulent intensities at Re = 0.36 (1 ml/h), see Supplementary Fig. S3. Note that the velocity profile for Re = 0.36 does not exhibit any central peak. This provides evidence that for the viscoelastic liquids, the observed peak at Re = 0.217 (De = 414.6, 10 ml/h) in Fig. 2 of the main manuscript was not due to fluid inertia. It should be noted that despite of a lower flow rate, the Reynolds number is larger for the Newtonian liquids which have a lower viscosity as compared to the viscoelastic test liquids.

Fluids Rheology

The rheological properties of the liquids under shear were measured with the Gemini Hr Nano rheometer, while the extensional relaxation tests were conducted with the Capillary Breakup Extensional Rheometer (CABER). Here, the Reynolds number (Re) is defined as $\mathrm{Re} = 2\rho\dot{Q}/\eta(w_c + d)$, where ρ is the liquid density, $\dot{Q}$ is the flow rate, η is the solution shear viscosity in the contraction, w_c is the contraction width occupied by each fluid, and d is the depth respectively. The Deborah number (De) is defined as $De = 2\lambda_e\dot{Q}/w_c d^2$, where λ_e is the extensional relaxation time. As an approximation, elastic effects were neglected in the numerical simulations with COMSOL Multiphysics, which showed that the main-stream liquid occupies about 44% of the cross-sectional area in the contraction. The viscosity of the liquid at the

specific shear rate was then obtained from the rheological measurement, and used to compute Re. Due to the shear-thinning nature of the liquid, Re is not directly proportional to the flow rate. Supplementary Table S1 shows the rheological properties and corresponding Re and De for the experimental flow conditions.

PIV Measurements

For PIV measurements, the Nikon B-2A filter was removed and white light illumination was used. Polystyrene particles (5 µm, Thermo Scientific) were seeded to the liquids (0.1% w/w) to facilitate the PIV measurements. Short exposure times of 2 µs were used to preserve image sharpness. Each interrogation window was 64 x 32 pixels, with a 50% overlap between consecutive windows. The recording rates at De = 41.46 and De = 414.6 were 250 FPS and 2000 FPS respectively. Based on a cross-correlation algorithm in MATLAB, the resulting velocimetry data were computed. To obtain meaningful average velocity profile over a longer time span, the recording rates were lowered to 100 Hz for capturing 10,000 image pairs i.e. a time interval of 10 ms from one image pair to the next pair. The time interval between the two images in each pair was 4 ms and 0.5 ms, at De = 41.46 and De = 414.6 respectively. These allowed the computation of the normalized mean axial velocity profiles ($\overline{U}_i / \overline{U}_{bulk}$) and turbulent intensities ($u_{i,rms} / \overline{U_i}$) across the channel width (1 mm) at δ = 0.3 mm, as shown in Fig. 2 of the main manuscript (for definition of δ, refer to Supplementary Fig. S1).
